# COVID-19 Infodemic: Evaluating Information-Seeking Behaviour Among Healthcare Workers During a Pandemic

**DOI:** 10.7759/cureus.20910

**Published:** 2022-01-03

**Authors:** Raman Sharma, Mahendra Kumar, Kusum K Rohilla

**Affiliations:** 1 Hospital Administration, Postgraduate Institute of Medical Education and Research, Chandigarh, IND; 2 Nursing, Postgraduate Institute of Medical Education and Research, Chandigarh, IND; 3 College of Nursing, All India Institute of Medical Sciences, Rishikesh, Rishikesh, IND

**Keywords:** pandemic, healthcare workers, information seeking behavior, covid-19, infodemic

## Abstract

Introduction

With the surge in coronavirus disease 2019 (COVID-19) cases across nations worldwide, World Health Organization (WHO) declared it a pandemic on March 11, 2020. Besides various policy guidelines and directions issued from time to time to combat the menace, there was the dissemination of a lot of misinformation and disinformation through social media or otherwise. This led to emotional, psychological, and mental agonies in the general population as well as Healthcare Workers (HCWs).

Methods

A cross-sectional quantitative study using purposive sampling techniques was done to assess the health-seeking behavior during the pandemic among HCWs of a tertiary care hospital in North India, designated as a dedicated COVID-19 hospital by the Ministry of Health and Family Welfare, India. A semi-structured questionnaire validated by experts with a reliability value (r=0.92) was taken. To maintain minimal contact and to follow COVID-19 Appropriate Behavior (CAB), a web-based link was used to obtain the data. Privacy of the participants and confidentiality of data obtained was maintained.

Results

Out of the total 250 participants enrolled in the study, the response rate was 81% (203). For 123 (60%) respondents, social media was one of the sources of access to information during the pandemic. The most common social media app(s) accessed were WhatsApp 82 (40%), Facebook, and Instagram 67 (33%). Social media had both positive as well as negative impacts on the mindset of respondents as 147 (72%) agreed that social media networks helped to further improve their understanding, whereas 178 (88%) stated that it aroused fear and panic among them.

Conclusion

During epidemics, timely, accurate, and authentic information is vital in shaping public opinion; on the contrary, an infodemic can pose a serious threat and panic in society by disseminating false and wrong information as was seen in the COVID-19 pandemic.

## Introduction

Since the identification of the first case in Wuhan, China, in December 2019 and the rapid surge of coronavirus disease 2019 (COVID-19) in more than 200 countries, the World Health Organization (WHO) declared it a Public Health Emergency of International Concern (PHEIC) on March 11, 2020 [1,2]. To combat this rise in cases, various directions and guidelines were issued from time to time by different national and international agencies along with the imposition of strict restrictions and lockdowns affecting routine life worldwide [3]. Besides this, there was also dissemination of lots of misinformation and disinformation through social media or otherwise, which had led the people in the lurch to whom to rely on or not. This has led people to experience a lot of emotional disturbances leading to stress, panic, and fear as well as mental health disorders like depression and anxiety.

At the 2020 Munich Conference, the WHO chief stated that "we are not just fighting a pandemic; we are also fighting an infodemic," which has highlighted the risk of fake information being disseminated through these social media networks [4]. Social media has been blamed for spreading fear and panic among people as some nasty players tried to fulfill their agendas by expressing their views and unconfirmed and fake news through social media [5], From the outset of the pandemic, Indian government agencies have found an alarming number of fake news stories associated with COVID-19 circulating on social media [6]. So, the need of the time is to have international cooperation and coordination amongst communities and nations worldwide, collective efforts to disseminate authentic, accurate, timely useful information for community awareness, treatment collaboration, and research which will aid in fighting future outbreaks effectively.

It is well-known fact that with the technological advancements of the 21st century, social media has billions of users globally and according to a study, there is an extreme rise in social media users [7,8]. The power of social media can be understood by recent reports that claim that by the end of the year 2022 approximately 3.29 billion social media users will be available worldwide, which covers around 42% of the entire world’s population [9,10]. In the present scenario, major platforms of social media being explored include Facebook, Instagram, Whatsapp, Twitter, and so on. A UK-based study has shown that 55% of global internet users are on social media platforms, viz. Whatsapp, Facebook YouTube, and Twitter [11]. Whatsapp alone has more than 900 million users across India [12]. Hence, with this large chunk of users across the world, one click can disseminate any information or news, right or wrong, good or bad, in any corner of the world within a fraction of a second. Even more, people nowadays use social media as a first-hand information source to gain information about COVID-19 [13]. Many studies have supported the fact that social media has played an important role in disseminating information and awareness about the COVID-19 pandemic among people [9,14,15]. With this background, the present study was conceived with the objectives to assess the information-seeking behavior amongst HCWs on social media during the COVID-19 pandemic. 

## Materials and methods

This was a cross-sectional quantitative study conducted in one of the premier institutes of North India, Postgraduate Institute of Medical Education and Research, Chandigarh, designated as a dedicated COVID-19 hospital by the Ministry of Health and Family Welfare (MOHFW), India. To assess the health-seeking behavior among HCWs during the pandemic, a semi-structured questionnaire validated by experts from the field of Internal Medicine and Community and Family Medicine was developed, having a reliability value (r=0.92). A purposive sampling technique was followed and a sample size of 250 HCW (specifically those who have worked for the management of COVID-19 patients admitted in the COVID-19 hospital) were enrolled. To maintain minimal contact and follow COVID-19 appropriate behavior (CAB), a web-based link was used among the study participants to obtain the data.

The questionnaire was prepared in English, with two sections; Section-I included a demographic profile of the participants whereas Section-II included questions in six domains, i.e. social media preferred apps, total time consumed on social media each day, and the type of information searched on social media. Information pertaining to which social media platform was used most during the COVID-19 pandemic, how did users decide to share the received content on a social media platform, and what influenced them most were sought (See Appendix).

Ethical approval was obtained from the Institute Ethics Committee, Postgraduate Institute of Medical Education and Research, Chandigarh (NK/6350/Study/487). Participants saw the study information and had to acknowledge the consent form before getting access to the questionnaire. Privacy of the participants and confidentiality of data obtained was maintained.

Data analysis was done by using IBM SPSS Statistics for Windows, Version 23.0 (released 2015, IBM Corp., Armonk, New York). Descriptive statistics were used to analyze the responses obtained.

## Results

**Figure 1:**
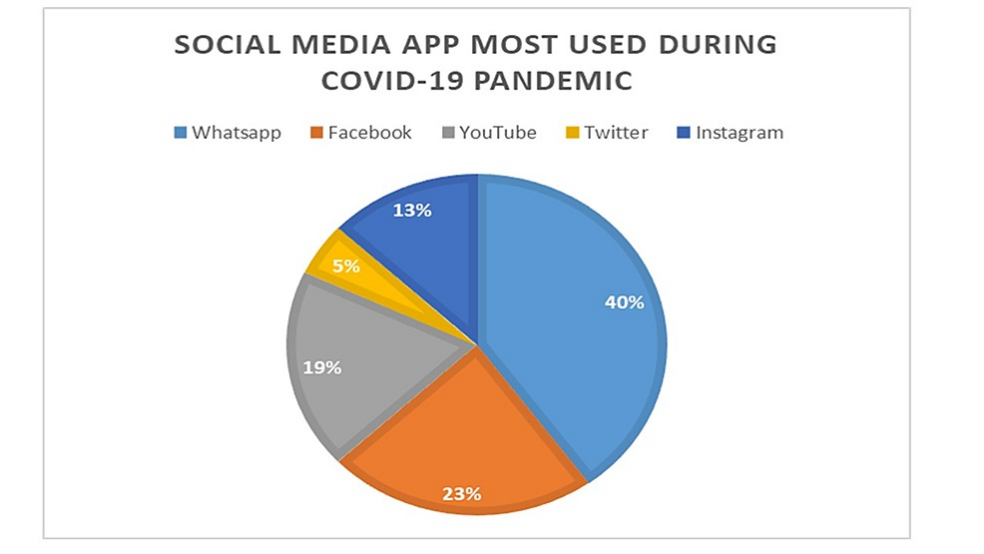
Social media applications most used during COVID-19 pandemic (n=203)

Out of the total 250 participants enrolled in the study, the response rate was 81% (203). And, out of the 203 respondents, the majority (n=84, 41%), were in the age group of 18 to 25 years; most of the respondents were female (n=171, 84%). As no newspaper(s) were being distributed during the COVID-19 pandemic peak, 123 (60%) respondents stated that social media was one of the sources to have access to information about the ongoing pandemic. It was further found that out of the available social media networks, the most popular social media app(s) were Whatsapp (n=82, 40%), Facebook and Instagram (n=67, 33%), followed by YouTube and Twitter, which were used by 41 (20%) and 33 (16%), respectively (Figure 1). Around 90 (44%) respondents stated that they used to spend two to three hours approximately per day surfing these apps.

The news items most often accessed were related to COVID-19, viz. surge in the number of COVID-19 cases (n=82, 40%), failure of medical facilities like sudden oxygen scarcity, bed shortages in hospitals due to a rapid surge of patients (n=74, 36%), COVID-19 mortality news (n=68, 33%), and COVID-19 vaccination (n=69, 34%). Other news was also accessed by 108 (53%) respondents. As per the findings, social media had both positive as well as negative impacts on the mindset of respondents as 147 (72%) agreed that COVID-19 news disseminated through social media networks helped to further improve their understanding of COVID-19, whereas 178 (88%) were of the view that it aroused fear and panic among them (Table 1).

**Table 1:** Information-seeking behaviour of health care workers during COVID-19 pandemic (N=203)

S. No.	Variables	Options	f (%age)
1	Topic most seen/read/heard on social media during the COVID-19 pandemic	Surge in COVID-19 news	82 (40)
		Medical facilities failure news	74(36)
		COVID-19 patients’ mortality news	68(33)
		COVID-19 vaccine news	69(34)
		Other than COVID-19 news	108(53)
2	Use of social media platforms during COVID-19 helped people	Increasing knowledge about safety from COVID-19 pandemic	99(49)
		Passing crucial information about COVID-19 among public	41 (21)
		Helping the public through social help support groups	32 (15)
		Helping people find out the availability of health care facilities nearby	32 (15)
3	COVID-19 news on social media spread fear and panic among people	Yes	189 (88)
		No	24 (12)
4	Information that disturbed most on social media during COVID-19 Pandemic	Excessive coverage of news related to COVID-19 mortality	104 (49)
		Excessive coverage of panic-inducing information related to COVID-19 infection surge	93 (45)
		Excessive coverage of videos, photos, and news of the countries with a high number of COVID-19 cases	87 (42)
		Excessive coverage of UNConfirmed news about the possibility of another COVID-19 outbreak in the near future	75 (36)
		Nothing as such disturbing	109(53)
5	Social media platforms have significantly contributed to changes in behaviour to prevent COVID-19 by taking various preventive measures	Yes	164 (80)
		No	10 (5)
		No response	29 (15)

**Figure 2:**
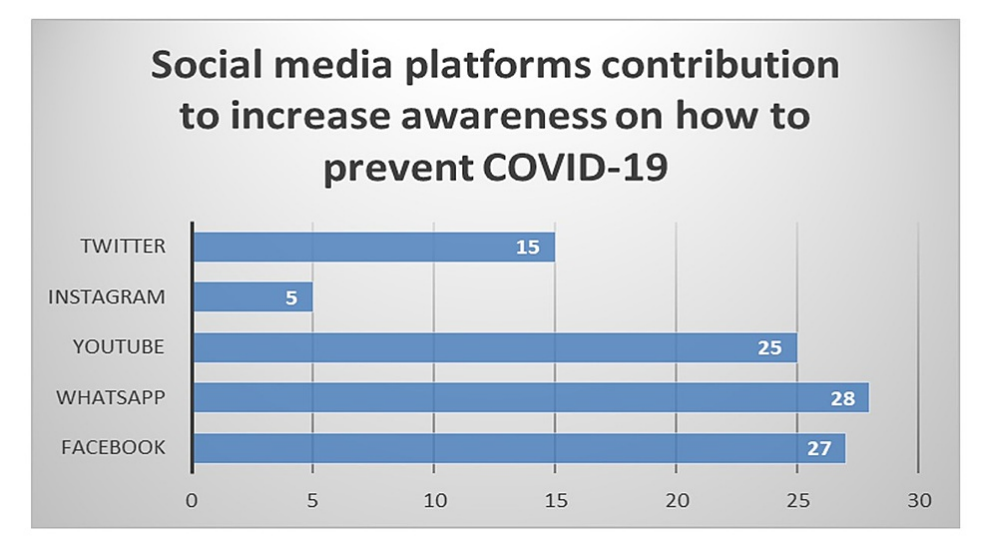
Usage of social media platforms to gather information on how to prevent COVID-19

As per the responses, 99 (49%) think that these social media networks were helpful in improving knowledge and awareness about safety measures to be taken during the pandemic, 41 (21%) think they help in passing crucial information about COVID-19 among the public, 32 (15%) are of the view that social media was helping public through social help support groups, and 31 (15%) feel that they were helping people to find out the availability of nearby health care facilities. Importantly, 112 (55%) respondents agreed that these social media platforms had significantly contributed to changes in their behaviour to prevent COVID-19 by taking various preemptive measures. Out of these various available social media platforms, WhatsApp was used the most (n=86, 42%), to improve awareness about COVID-19. On the contrary, these also led to arousal of panic and fear among respondents through excessive coverage of news related to COVID-19 mortality (n=104, 49%), coverage of panic-inducing information related to COVID-19 infection surge (n=93, 45%). Most of the participants said that WhatsApp (28%) was the most dominant social media platforms contribution to increase awareness on how to prevent COVID-19 (Figure 2).

Of the respondents, 154 (76%) stated that similar or same messages/information came from different sources repeatedly. Further, the majority of respondents (n=81, 40%) stated that that they never forwarded messages related to the COVID-19 outbreak at all, while 25 (12%) stated that they forwarded without even reading the subject matter of the message received (Table 2).

**Table 2:** Reaction of participants on receipt of messages related to COVID-19 (n=203)

S. No.	Variables	Options	Frequency (Percentage)
1	Received same messages/ information repeatedly related to COVID-19	Yes	154 (76)
		No	49(24)
2	What I did whenever I received messages via social media related to COVID-19 outbreak	I do not forward any messages at all	81 (40)
		I read the messages, and then forwarded it	48(24)
		I forwarded after a while if I know the source of information	49(24)
		I forwarded the messages directly	25(12)

## Discussion

During the COVID-19 pandemic, social media played a vital role in the dissemination of information about the virus, including various guidelines and policies issued from time to time for information and awareness among HCWs as well as the general population. Various HCWs have also been recognised and appreciated as heroes or ‘Corona warriors’ worldwide through social media coverage [4].

On the other side of the coin, the same media networks have made HCWs face challenges with the flashing of misinformation and disinformation, thereby leading HCWs to suffer from stress, depression, and anxiety. In the present study, 123 (60%) respondents stated that social media was a vital link to access information about the ongoing pandemic as well as other news and narratives. Similarly, another cross-sectional study from the Indian subcontinent showed that during the COVID-19 pandemic lockdown, approximately 42% of study participants reported frequent use of mobile phones to talk to their family and friends as compared to 33% before the lockdown [14]. During the peak of the COVID-19 pandemic, people have been using social media since they found it handy and quicker to get and share information related to treatment, bed availability in nearby hospitals, medicine stores, and other vital information related to COVID-19 treatment and prevention [12]. Similarly, another study also reported social media as an important source for finding health information as well as a major platform for sharing personal experiences, opinions, and concerns about health, illnesses, and treatment [16]. Further, over 44% of study respondents accepted that they used to spend approximately two to three hours per day on social media surfing information about the pandemic. The findings were similar to another study that stated a 50% rise in surfing time during the COVID-19 pandemic [17]. A study by Kalyani et al. showed that participants spent over half an hour searching for information related to COVID-19 each day during the pandemic [18].

In the present study, respondents have stated that these social media networks were very helpful in different aspects, viz. improving their knowledge and awareness about safety measures to be opted during the pandemic (n=99, 49%), passing crucial information about COVID-19 among the public (n=41, 21%), helping public through social help support groups (n=32, 15%), helping people to find out the availability of healthcare facilities nearby (n=31, 15%), which has significantly contributed to changes in their behaviour to prevent COVID-19 by taking various preemptive measures. These findings are similar to that of another study by Li and Liu in China, where the participants found social media to be the first choice for getting news and information about COVID-19. They found social media to be the highest predictor of preventive behaviour. [19]. On the contrary, various studies have suggested that users spend more time on negative news related to the COVID-19 pandemic, which has a higher risk of impacting one’s mental health [4,5]. Social media played a major role in creating panic and fear among users by spreading rumours and fake news [4]. These findings are similar to that of the present study where 49% of study participants agreed that they got disturbed and panicky by excessive COVID-19 mortality-related news [20].

In the current study, we found that WhatsApp was the most preferred social media platform for information during the pandemic and the reason for this may be due to the WhatsApp having the highest number of users along with Facebook [6]. A study by Galhardi et al. also reported that fake news seen on different social media platforms was mainly shared on WhatsApp [21], although there is a need to do more work to examine which social media app or platform was producing the highest fear and misinformation during the pandemic. Also, it is difficult for responsible agencies to counter wrong information being spread by users on these platforms as any user can post information without any credible source [16]. Here, Twitter has been an exception as most government agencies have official accounts on the platform and could clarify any rumours and misinformation within a safe time [22].

Various studies across the world have reported that there has been a sharp rise in fake news about COVID-19 [3,12,23]. More studies have reported that as much as 25% of the COVID-19 related information circulating on social media platforms, may contain some amount of disinformation [13,24]; after the intervention of WHO, a few social media platforms have removed unconfirmed content from their platforms [25]. The information shared by users on social media platforms can be subjective, right or wrong, misinformation, disinformation, and/or conspiracy theories [23]. The constant, uncontrolled flow of deceptive information on social media extends confusion and turns a pandemic into an infodemic [26]. An important observation from the current study was that 76% of respondents accepted that they were receiving the same messages from different users and the majority of respondents stated that they do not forward messages related to the COVID-19 outbreak at all without confirming the source of information. Only 12% of study participants reported forwarding social media messages without confirming the sources. This shows the good impact of induction training given to nurses before they were being posted to COVID-19 designated units.

## Conclusions

With technological advancements, social media has an extremely high number of users globally. People very often use social media for seeking health information and this same social media may also lead to crisis-like situations due to too much information or an infodemic. The goal of the study was to determine how HCWs used social media during the COVID-19 pandemic, the effects of social media information overload about COVID-19, and its long-term impact on the mental health of healthcare workers. WhatsApp was seen to be the most preferred social media platform for information during the pandemic. An important observation was that although 76% of respondents said that they received the same messages from different users, the majority of respondents stated that they do not forward messages related to the COVID-19 without confirming the source of information. There is an urgent need for more detailed research on the effects of COVID-19 infodemic and its long-term impact on the mental health of HCWs.

## Appendices

**Table 3:** Questionnaire for assessing information-seeking behaviour of health care workers

S. No.	Variables	Options	Ranking (1^st^/2^nd^/3^rd^/4^th^)
1.	Topic most seen/read/heard on social media during the COVID-19 pandemic	Surge in COVID-19 news	
		Medical facilities failure news	
		COVID-19 patients’ mortality news	
		COVID-19 vaccine news	
		Other than COVID-19 news	
2	Use of social media platforms during COVID-19 helped people	By Increasing knowledge about safety from COVID-19 pandemic	
		By passing crucial information about COVID-19 among public	
		Helping public through social help support groups	
		Help people to find out availability of health care facilities nearby	
			Yes/No
3	COVID-19 news on social media spread fear and panic among people	Yes	
		No	
			Ranking (1^st^/2^nd^/3^rd^/4^th^)
4	Disturb most on social media during COVID-19 Pandemic	Excessive coverage of news related to COVID-19 mortality	
		Excessive coverage of panic-inducing information related to COVID-19 infection surge	
		Excessive coverage of videos, photos, and news of the countries with a high number of COVID-19 cases	
		Excessive coverage of unconfirmed news about possibility of another COVID-19 outbreak in near future	
		Nothing as such disturbing	
			Yes/No/NA
5	Social media platform has significantly contribute to changes in behaviour to prevent COVID-19 by taking various preventive measures	Yes	
		No	
		No response	
			Yes/No
6	Received same messages/ information repeatedly related to COVID-19	Yes	
		No	
			Ranking (1^st^/2^nd^/3^rd^/4^th^)
7	What I did whenever I receive messages via social media related to COVID-19 outbreak	I do not forward any messages at all	
		I read the messages, and then forwarded it	
		I forwarded after a while if I know the source of information	
		I forwarded the messages directly	
